# Practical Use of Robot Manipulators as Intelligent Manufacturing Systems

**DOI:** 10.3390/s18092877

**Published:** 2018-08-31

**Authors:** Pablo J. Alhama Blanco, Fares J. Abu-Dakka, Mohamed Abderrahim

**Affiliations:** Department of Systems Engineering and Automation, University of Carlos III, Escuela Politécnica Superior, Avd. de la Universidad 30, 28911 Leganés, Spain; fabudakk@ing.uc3m.es (F.J.A.-D.); mohamed@ing.uc3m.es (M.A.)

**Keywords:** industrial internet, intelligent manufacturing, Industry 4.0, toolbox, smart factory, multi-physical systems, big data, intelligent manufacturing core (IMC)

## Abstract

This paper presents features and advanced settings for a robot manipulator controller in a fully interconnected intelligent manufacturing system. Every system is made up of different agents. As also occurs in the Internet of Things and smart cities, the big issue here is to ensure not only that implementation is key, but also that there is better common understanding among the main players. The commitment of all agents is still required to translate that understanding into practice in Industry 4.0. Mutual interactions such as machine-to-machine and man-to-machine are solved in real time with cyber physical capabilities. This paper explores intelligent manufacturing through the context of industrial robot manipulators within a Smart Factory. An online communication algorithm with proven intelligent manufacturing abilities is proposed to solve real-time interactions. The algorithm is developed to manage and control all robot parameters in real-time. The proposed tool in conjunction with the intelligent manufacturing core incorporates data from the robot manipulators into the industrial big data to manage the factory. The novelty is a communication tool that implements the Industry 4.0 standards to allow communications among the required entities in the complete system. It is achieved by the developed tool and implemented in a real robot and simulation

## 1. Introduction

The concept of Industry 4.0 is broad and potentially ambiguous. Industry 4.0 presents several difficulties regarding the nature of the actual concepts of intelligent manufacturing including the inexorable competition that exists on the global market, with its changing social, economic and political structures of the industrial sphere. In practical terms, this necessitates a technological boost in industrial practices [1,2]. This technological spur is the most direct effect of using manufacturing robots in industry, and this last aspect has a direct impact regarding robot manipulators used in the industry.

Each element in a supply chain management must interact and communicate with the other elements in an efficient and coordinated manner. Robot manipulators are widely used in manufacturing, e.g., palletizing robots where offline-programming (OLP) [3,4] is required. This OLP makes the interaction with the robot impossible from the cyber-physical system point of view and creates a barrier to intelligent manufacturing [5]. Logistical problems, payment method security or products that are difficult to trade online can all be barriers to e-commerce [6]. In literature, some researchers propose combining a web service with a humanoid robot [7]. For educational and research purposes, researchers developed client applications for experimentation to increase the efficiency [8]; however, these applications did not comply with Industry 4.0 standards. Moreover, in an effective manner, emerging information systems are being developed to integrate multiagent systems (MAS) with Service Oriented Architectures (SoA) [9], but failed to refer to the practical reality in their manufacturing industry. External interfaces are used to connect the production system with the surrounding production site, mainly with systems for user interaction as human machine interfaces (HMI), machine and production data acquisition or production management as machine execution systems (MES) [10]. It is now at the stage where it must transform those concepts into an operational reality.

The tools needed to fully integrate a robot manipulator in the Smart Factory as a piece of Robotic Innovation Facility (RIF) equipment must operate within Industry 4.0’s scope. Our proposed toolbox has been created under the Industry 4.0 philosophy to operate in an industrial case study: a bin-picking of objects jumbled together inside a big basket. The work setting has been documented within the manufacturing process and selected using an analytical hierarchy process in accordance with Industry 4.0 criteria, taking into account the company’s strategic objectives and the feasibility of its implementation. The work setting is summarized in Section 5.

**Figure 1:**
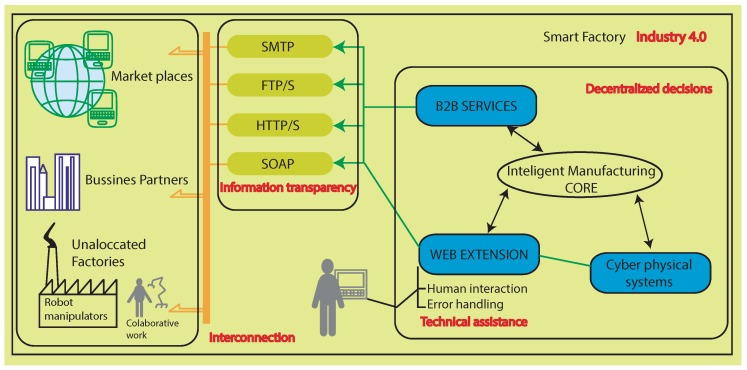
Principle scheme that shows the intervening agents in a process from a purchase, going through a decentralized decision-making until the unallocated manufacturing.

The sequence followed by the case study within intelligent manufacturing is part of either the business to client (B2C) or business to business (B2B) process that starts from a customer or company that wants to buy, for example, a product of the series 1 that contains products of type A and type B. Either through marketplaces or a business partner, the requirement for the product is defined and the robot manipulator is prepared for the specific task of series 1. In other words, while the robot performs a given task(s), it receives information about new one(s). Whether it will be type A or B needs to be defined by the client at this juncture. Moreover, depending on the delivery period, their manufacture is prioritized over others with longer lead times. At this moment, the product to be manufactured is known by the Intelligent Manufacturing Core (IMC) of the manipulator and, therefore, the task to be performed. However, the process will wait for the confirmation of the order or any other requirements to validate the manufacturing task. The complete principle scheme can be visualized graphically through Figure 1, as in a detailed description in Section 5.

The novelty is a communication tool that implements the Industry 4.0 standards to allow communications among the required entities in the complete system.The tool developed is intended to simplify the use of robot manipulators inside a smart factory with the warranty of Industry 4.0 compliance. It allows a direct interconnection between robot manipulator in allocated factories, business partners, market places and error handlers by transparency information support in conjunction with the IMC allowing for making decentralized decisions. The advantage of all these kinds of characteristics together resides in a easy implementation with a high potentiality of use and serves as an example for other actors working in relation with a smart factory.

The rest of the paper is structured as follows: The “web services” related to industrial manipulators are covered in Section 2. Section 3 focuses on Industry 4.0 rules that may apply to modern industry. A description of the tool that will integrate and control the robot in the smart factory in real time or simulation is presented in Section 4. In Section 5, we present our case study and show how it complies with Industry 4.0 requirements. We valuate our approach in both simulation and real scenario as described in Section 6. Finally, results, conclusions and future research are given in Section 7 and Section 8.

## 2. Web Services for Manipulator-Like Devices

The term web services encompasses a wide range of products and services with Internet support. A practical approach to intelligent manufacturing requires that all devices have network-enabled remote access capability and that they can interact with other devices, databases or peripheral equipment, thus enabling a more efficient use of this equipment. There are different communication protocols and languages available for real-time use:

(I) Simple Object Access Protocol (SOAP);
(II) Representational State Transfer (REST);
(III) JavaScript Object Notation (JSON);
(IV) Web Services Description Language (WSDL);
(V) Web Application Description Language (WADL);
(VI) Universal Description, Discovery and Integration (UDDI);
(VII) Windows Communication Foundation (WCF).

Four of the leading manufacturers of industrial manipulators are discussed below with examples of implementations and data provided by the manufacturers themselves.

### 2.1. ABB

It is one of the leading manufacturers of industrial robot manipulators and has incorporated Robot Web Services starting from version 6.0 of its RobotWare software (Västerås, Sweden). Specifically, it uses a REST API (Application Programming Interface) by applying HTTP methods that react in Extensible Markup Language (XML) or JSON. The client application does not need specific licensing or libraries to use this manipulator and works directly on the controller, regardless of whether it be real or virtual. The API supports the following services:

- Get RobotWare services,
- CFG Service (Read/write configuration’s data),
- DIPC Service (Distributed Inter-Process Communication),
- Event log Service,
- IO Service (Read/write/subscribe IO-signals, IO-devices and IO-networks),
- Mastership Service,
- Panel Service,
- RAPID Service (Read/write/subscribe on rapid variables. Load/unload program/modules, Start/stop program.),
- System Service,
- RobotWare return codes service.

### 2.2. FANUC

Without going into detail, FANUC (Rochester Hills, MI, USA) controllers are not based on a PC architecture and, even though subject to their own patent system, they are available for use through webservices as can be seen in [11,12], where a module is implemented to ensure the communication with the controller so that the latter can enable the web service. While it is true that they are separate instances, they do not appear to be easily integrated into an intelligent manufacturing set-up.

### 2.3. KUKA

“Robotic as a Service” is the term used by the Lead Architect Industry 4.0 at KUKA(Gersthofen, Germany) which offers a Robot controller with a Service-Oriented Architecture (SOA) server offering “motion services” as a solution [13]. This concept was already presented in [14] where the authors related it to cloud computing. In particular, KUKA.Ethernet RSI XML and KUKA.Ethernet KRL XML are Application Programming interfaces that are used for point-to-point communication at either a cycle of 12 ms or that is non-cyclic (on demand).

### 2.4. Stäubli CS8C

Some of the leading robot manipulator manufacturers do not clearly and openly market the web services for their industrial robots. This is true of this particular manufacturer. Their service is semi-hidden, poorly documented and only accessible via the manufacturer. Their controllers have a Low Level Interface (LLI) for developing low-level control applications in position and speed modes, as well as in torque control mode. One drawback of this low level is that all the advanced features of a high-level controller that are used in industry are lost, and these features affect certain aspects that are as important as safety limitations or even the kinematic control itself. The situation of our case study presented in Section 5 requires direct communication with the industrial manipulator, in order to allow direct remote control without specifically pre-programming the controller.

The controllers of industrial robot manipulators are based on real-time systems, such as the high-performance real-time operating system VxWorks. The driver cycle times are shown in Table 1. A SOAP server that uses an XML message exchange protocol on the network is enabled on the real-time system.

## 3. Current Industry 4.0 Requirements

This section clearly sets out the Industry 4.0 principles faced by industry today. The different problems that need improvement and integration that are facing manufacturers include the multitude of systems, the diversity of specific machinery for each task, the lack of communication between them all, Enterprise Resource Planning (ERP) managed maintenance, the absence of a Manufacturing Execution System (MES) in all the machines, and the concept that the geographical location of a factory should not affect its integration in the system [15], among others.

### 3.1. Interconnection

This is one of the fundamental design principles that should allow total interaction with the rest of the systems active in Industry 4.0.

### 3.2. Information Transparency

This is the tool that will allow data collection from the physical world by the robot manipulator in real time. It will also make the data available to the smart factory for intelligent decision-making in the other systems. It should be endowed with a cyber physical nature [16] that allows it to be used in a simulated environment where digital information models become virtual copies of the physical world [17].

### 3.3. Decentralized Decisions

This feature is derived from requirements A and B. Data interconnection plus information transparency should create an ecosystem that allows decentralized decision-making. All the relevant data of the industrial robot manipulator should be accessible [18]. This would allow a financial or logistic parameter that is outside the production line to directly modify the production task performance in real time and receive useful instant informative feedback for the decision-making process. Conflicts or exceptions are the field of a wider concept of Technical Assistance.

### 3.4. Technical Assistance

It must be possible to assist the robot manipulator when its decentralized decision-making cannot resolve the problem automatically. Technical assistance must also be possible when necessary due to the complexity of the production process so human–machine interaction [19] must be possible whenever human intervention is required. This is known as error handling and includes strict compliance with minimum safety directives to guarantee machine or process starting by the voluntary activation of manual controls designed for this purpose. Error handling must be possible even in other areas in which the smart factory is unable to make a decision on its own, for example, in the economic field.

## 4. Tool Description

This section presents a method of direct remote access to the controller that works by creating classes using WSDL in Matlab^®^. It allows 4.0 integration with functions for robot data acquisition and online control, thereby allowing users to monitor, analyze and even control the robot in real time using the methods implemented in our Toolbox. SOAP provides a method of accessing remote objects through XML messages regardless of the platform and language employed. It works on various low-level communication protocols.

The controller’s different SOAP versions have evolved and improved over time even to the point where more advanced versions can change the program execution pointer. The first version 5.3, uses a single server. It offers identification methods like “find” or “ping”, user validation including “login” and “logout” and can obtain information about the number of robots connected to the controller, Cartesian and articular positions, or basic controller parameters. A proof of the reliability and evolution of the service is its improvement over time with the incorporation of new methods. The service has increased its reliability and incorporated new methods. For instance, beginning with version 7, the service has been constituted by four servers. The new methods not only allow access to information, but also allow real-time control of the robot and all its parameters, including modification of internal program variables or even full program loading. All of these methods run on the industrial controller that is operating at a high level like the high level language used by industrial controllers.

**Figure 2:**
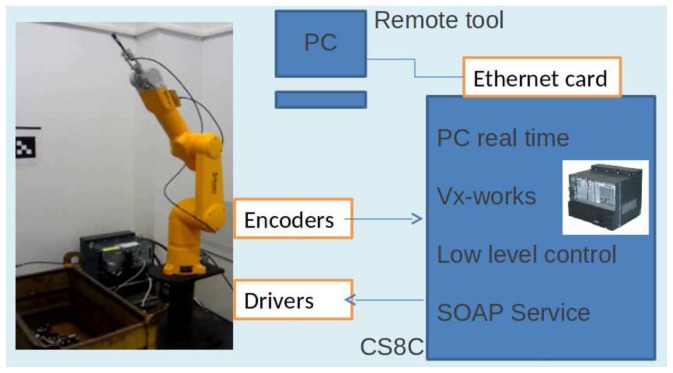
Configuration used on the real robot Stäubli TX 60 L (Stäubli AG, Horgen, Switzerland) and its controller CS8C.

Our new toolbox allows real-time remote control. The greatest advantage of the protocol used is that it works correctly on network firewalls, differently from other web service formats. The components of the system are shown in Figure 2.

Briefly, for each message, this tool accesses remote objects through SOAP XML messages. Regardless of the platform and the language used, the methods run on the controller at a high level in a manner that is compatible with the proprietary languages of each manufacturer. At a low level, the message structure consists of eight well-differentiated parts.

1. **The HTTP request header** is the first part of the message or heading.
2. **The SOAPAction header** is the header for firewalls or the network infrastructure mainly responsible for filtering and routing. The value is a uniform resource identifier (URI).
3. **The SOAP envelope** is the HTTP request that contains the SOAP request itself. It contains metadata important to understanding the request. The SOAP-ENC: encodingStyle attribute is the URI that indicates the structure of the body of the message.
4. **SOAP encodings** is the method for structuring the body of the message, i.e., the code used for SOAP serialization.
5. **The SOAP body** is another layer that encloses the current elements of a given event, specifically those elements that correspond to the login. The body clearly marks the separation between data and metadata.
6. **HTTP Response header** is the equivalent heading in the response.
7. **The return result** in the interface definition language (IDL) can give a “void" value, which means that there is no response.

## 5. Case Study and Appropriateness to Industry 4.0

This section shows a case study that could set up a smart factory. The factory is presently selecting and then distributing castings using mechanical gravity vibrators [20,21] and the owners plan to adapt the process to Industry 4.0. The process begins when a customer who is finalizing their online order selects the desired piece. At this time, the characteristics of the piece have already been defined and planning can be started in advance. The anticipatory capacity of the system that already exists in the ordering phase makes it possible to reduce processing times on the production line.

The task is fully automated with a real-time response of picking the selected parts. In addition, all available information related to the piece itself is also transmitted as well as the process activities like piece characteristics verification, for instance weight, the times of use, unscheduled stops and maintenance, etc. Specific components involved in the task are shown in Figure 2, whereas Figure 1 shows aspects related to Industry 4.0.

### Case Study, Task and Industry 4.0

The case under study is basically composed of three agents involved in improving product supply chain performance [22]—first are the two aspects of the robot manipulator: the actual robot and the simulated robot. This duality provides the cyber/physical character [5]. The second agent is the interconnection network that links each part of the smart factory, bidirectionally and univocally, without forgetting the possibility for relocating each or any part or section, a possibility that introduces a paradigm of indirect costs totally different from the classic industrial model. Customer Relationship Management (CRM), Enterprise Resource Planning (ERP), Manufacturing Resource Planning (MRP), databases, etc., are all integrated in a smart factory [23]. Third, and no less important than the first two agents, is decentralized decision-making. Directly associated with our case study are two commonplace workplace situations. In the first, parts are manually selected by a worker [24]. In the second, vibratory part feeders sort the piece [20,21]. We propose a much more sophisticated third situation employing a sensorized industrial manipulator [25], coordinating this activity with the proposed Toolbox converts the task into a part of the smart factory.

Below, we present the process involved in the third setting as part of the scheme proposed in Figure 1.

#### Industrial Robot Manipulator

The real and/or virtual robot manipulator is transparently and remotely controlled in real time and integrated into the manufacturing process through its performance of regularly scheduled tasks.

#### Order

The order is received through the market place or business partners. The order will specify the units, model and color and other data.

#### Intelligent Manufacturing Core (IMC)

This is the decentralized decision-making core. Describing the full characteristics and potential of the IMC requires a deep analysis that is beyond the scope of the present article. Suffice it to say that, applying algorithms for artificial intelligence [26] and machine learning [27], the core can make autonomous decisions based on the information it has from all the agents associated with the business process, including the robot. This information processing is related with Big Data [28] and will not ignore the industrial robot manipulators. In fact, given its ability to physically interact with its environment, the robot is a key player in the industrial decision-making process. In summary, and in our example, the IMC receives the order, accesses the information from the supply chain, CRM, ERP, MRP, MES, etc., and makes the decision to stop the current task of the robot manipulator, which immediately starts working on the order based on criteria of profitability, customer attention or any other that has been defined and weighted by the company’s policy. The core remains active in an evolutionary monitoring mode in parallel to its intelligent decision-making.

#### Evolutionary Supervision Mode

The mode implements itself when an error occurs that has not been previously considered and human intervention is required to resolve it. An employee with decision-making ability determines the actions necessary to solve the error, either collaboratively with the robot or remotely following a procedure that ensures compliance with the minimum safety standards. Once the error is corrected, production is automatically resumed. The IMC will learn from the human intervention and will propose future decisions in accordance with the already-supervised solution.

## 6. Experiments

This section shows the behavior of the proposed tool for integrating the industrial manipulator within an intelligent production line. It also analyzes the level of integration in both the virtual and the real robot from the view point of Industry 4.0 design principles. The proposed tool is used to verify the real robot response in real time and show that the case study tasks were fulfilled in real time. Table 2 shows some of the methods used and Table 3 the performance reference [29].

**Figure 3:**
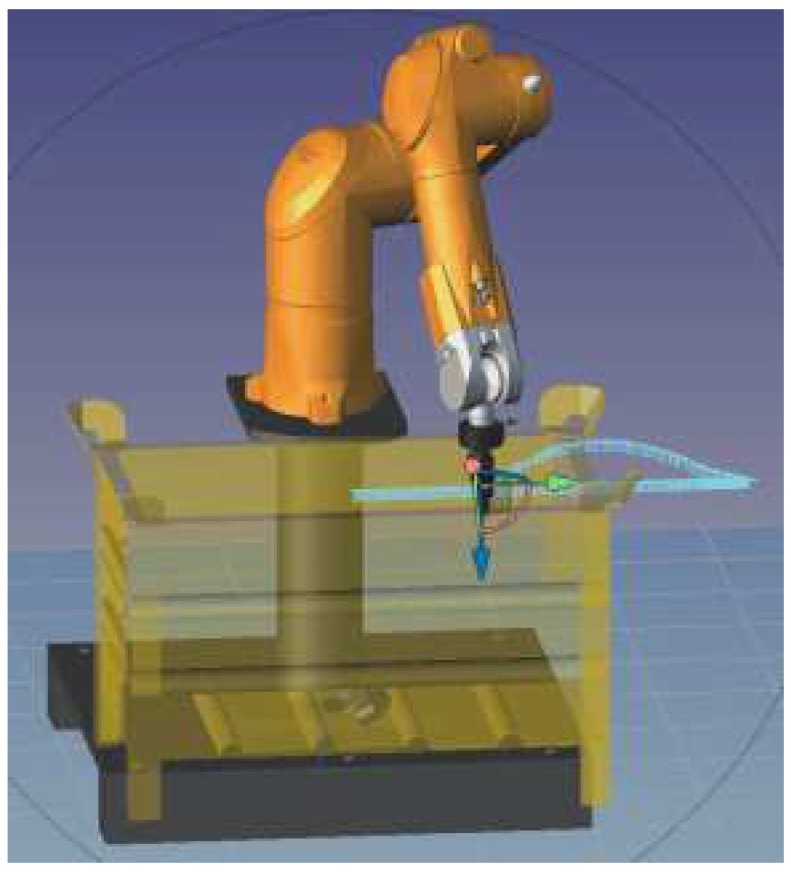
Simulation result of the use of the toolbox implementing obstacle avoidance on the Stäubli Robotics Suite (SRS) simulator. The straight trajectory represents the original trajectory free of obstacles in front of an adaptation online with the presence of a basket.

**Figure 4:**
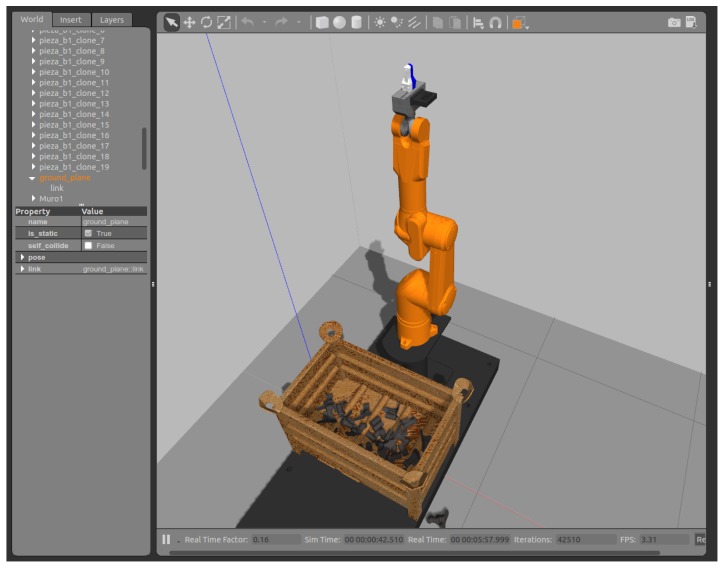
Simulation results of using the toolbox in a bin-picking task on the Gazebo simulator.

**Figure 5:**
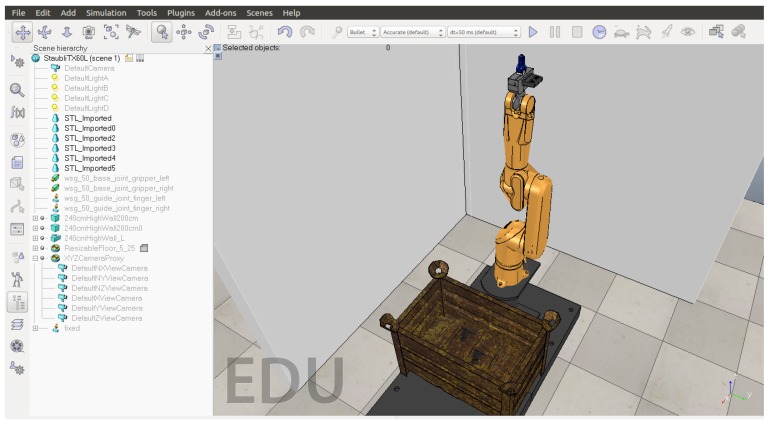
Simulation results of using the toolbox in a bin-picking task on the V-REP simulator.

The configuration shown in Figure 2 is valid for the actual controller and robot as well as for the virtual robots using the SRS software shown in Figure 3. It should be emphasized that the behavior of the simulation corresponds faithfully with the actual manipulator and facilitates rapid implementation in the real system. The configuration was also implemented in VREP 3.4 (Coppelia Robotics GmbH, Zürich, Switzerland) and GAZEBO 7.1 (Open Source Robotics Foundation, Mountain View, CA, USA) as can be seen in Figure 4 and Figure 5. They ran properly, demonstrating the versatility of this Toolbox.

Compliance with Industry 4.0 standards is shown below.

### 6.1. Interconnection

The IEEE 802.3 international standard is used in the industrial robot manipulator for the Stäubli CS8x controller. Its interconnective capacity makes it very simple to achieve integration. It is only necessary to integrate the controller into the network through its Internet Protocol address (IP), or automatic configuration can be triggered by activating the Dynamic Host Configuration Protocol (DHCP) on the controller.

### 6.2. Information Transparency

Toolbox interaction with the agents involved in the Smart Factory is quite transparent. The industrial robot manipulator receives messages from the IMC that are also sent to the virtual robot and integrated into the simulated virtual factory; robot information and parameters, such as cycle times, applied torques, power consumption, etc., are also shared. The versatility of the toolbox even allows its application within simulated environments like V-REP [30] or GAZEBO [31] or even in an industrial environment, as when the Stäubli Robotics Suite (SRS) is applied.

### 6.3. Decentralized Decisions

Each of the agents in the supply chain or business process exchange information with to the IMC. The variety of sources for information and data with a high decision value permits machine learning by the IMC, thus allowing evolutionary decision-making. The interconnections and the transparency of the information create an ecosystem that enables decentralized decision-making. The fact that there is access to all relevant data from the industrial robot manipulator allows a financial or logistic parameter, which may be external to the production line itself, to directly modify the execution of the production tasks in real time, and, also supplies feedback for instantaneous decision-making. In addition, human intervention is possible to enable the management of errors or exceptions that occur in the manufacturing process.

### 6.4. Technical Assistance

The controls in the Tool Box enables manipulator assistance when the decision-making process can not resolve a situation or the complexity of the production requires human interaction with the machine [19]. That is to say, all those situations in which human intervention is required are supported by the Toolbox, from error handling to decisions that the intelligent core cannot make by itself. The Toolbox has a web extension dedicated to handling errors or decisions derived from the IMC or even corrective maintenance tasks with human intervention.

## 7. Results

In the laboratory, the robot manipulator is controlled remotely as part of multi-physical or cyber-physical systems, such as multiple robots using real-time algorithms.

As an example of implementation, the developed tool was used to illustrate a Staübli TX60L manipulator remote supervisory control required for obstacle avoidance, as shown in Figure 3. The remote computer vision based supervisory system detects obstacles in the course of planed movement of the manipulator and instructs the robot to alter its trajectory accordingly through our Toolbox that communicates directly with the SOAP server in the controller of the manipulator. The nature of the target is irrelevant to our tool since it can work with either the real robot or with its virtual version (simulated). The tool is therefore so simple that it only requires changing the network configuration parameters to act transparently as an interface between any agent and the robot controller in order to integrate it in a smart factory setup. The computer vision system/software was provided by the industrial partner of the project and is beyond the scope of this paper.

The key results from the study are:

- The control algorithm admits intelligent manufacturing capabilities.
- It accesses online in real time to all of a robot’s parameters interchanging them with the whole system.
- The Industry 4.0 requirements are strict compliance. Interconnection, information transparency, decentralized decisions and technical assistance are supported.
- Introduces at a high level the main component of the Intelligent Manufacturing Core and the principle scheme.
- Allows several robot manipulators inside a smart factory to take action rapidly and secure a decision base in a transparency multi data environment.

## 8. Conclusions

The new industrial environment of interconnected mechanical systems is so highly competitive that intelligent, cooperative and dynamic manufacturing systems that can rapidly integrate the robot manipulators have become essential. The proposed tool can fulfill all the specifications of the design principles of the Industry 4.0. It creates a new environment/scenario allowing large scale access to the valuable data of the industrial manipulators on the part of any agent within the smart factory. This system also allows remote control of the robot manipulator itself. This can be done in real time or even through a virtual reality interface. Given the potential and need for these modalities, it seems reasonable to continue in the investigation and development of intelligent algorithms and tools that can round out the reach of the Intelligent Manufacturing Core (IMC) and manage the available big data generated within the smart factory.

References

## Figures and Tables

**Table 1:** Cycle times.

Option	Cycle Time [ms]	Value
by default	4	0.004
set 1	8	0.008
set 2	2	0.002
set 3	1	0.001

**Table 2:** Response times.

Methods	Response Time [s]
sid=login	0.009315
setPower	0.008121
resetMotion	0.006491
getApplications	0.008108

**Table 3:** Real-time requirements in different industrial environments [29].

Industrial Environments	Latency [ms]
Automation system	<50
Monitoring system	<100
Control operations	10–50
Motion control	<10
